# Hydrogel-Based Biomaterials Engineered from Natural-Derived Polysaccharides and Proteins for Hemostasis and Wound Healing

**DOI:** 10.3389/fbioe.2021.780187

**Published:** 2021-11-22

**Authors:** Junyao Cheng, Jianheng Liu, Ming Li, Zhongyang Liu, Xing Wang, Licheng Zhang, Zheng Wang

**Affiliations:** ^1^ Department of Orthopaedics, Chinese PLA General Hospital, Beijing, China; ^2^ Chinese PLA Medical School, Beijing, China; ^3^ Beijing National Laboratory for Molecular Sciences, Institute of Chemistry, Chinese Academy of Sciences, Beijing, China; ^4^ University of Chinese Academy of Sciences, Beijing, China

**Keywords:** hydrogel, hemostasis, wound healing, nature-derived polysaccharides, nature-derived proteins

## Abstract

Rapid and effective hemostasis is of great importance to improve the quality of treatment and save lives in emergency, surgical practice, civilian, and military settings. Traditional hemostatic materials such as tourniquets, gauze, bandages, and sponges have shown limited efficacy in the management of uncontrollable bleeding, resulting in widespread interest in the development of novel hemostatic materials and techniques. Benefiting from biocompatibility, degradability, injectability, tunable mechanical properties, and potential abilities to promote coagulation, wound healing, and anti-infection, hydrogel-based biomaterials, especially those on the basis of natural polysaccharides and proteins, have been increasingly explored in preclinical studies over the past few years. Despite the exciting research progress and initial commercial development of several hemostatic hydrogels, there is still a significant distance from the desired hemostatic effect applicable to clinical treatment. In this review, after elucidating the process of biological hemostasis, the latest progress of hydrogel biomaterials engineered from natural polysaccharides and proteins for hemostasis is discussed on the basis of comprehensive literature review. We have focused on the preparation strategies, physicochemical properties, hemostatic and wound-healing abilities of these novel biomaterials, and highlighted the challenges that needed to be addressed to achieve the transformation of laboratory research into clinical practice, and finally presented future research directions in this area.

## Introduction

Traumatic, surgical, disease-related, or drug-induced bleeding can lead to serious clinical outcomes or even death (Wang et al., 2019a). Traumatic blood loss caused by various sudden accidents (e. g. traffic accidents, natural disasters and violent attacks) is closely related to pre-hospital death. It is statistically estimated that about one-third of emergency deaths originate from acute blood loss and its secondary injuries (Kragh et al., 2012; Behrens et al., 2014), and more than half of the battlefield deaths are ascribed to massive blood loss (Hemostatic wound dressing, 2013). On the other hand, interventional diagnostic and surgical treatments during medical procedures are prone to hemorrhage or intracavitary bleeding, especially in areas adjacent to the heart, parenchymal organs, vital vessels, etc. Statistically, severe intraoperative blood loss directly contributes to increased mortality (Marietta et al., 2006). In addition, hematologic disorders or anticoagulant drugs are known to be responsible for abnormal coagulation, which likewise exposes many patients to high bleeding risks (Blajchman, 2003). Therefore, the exploration of rapid and effective methods for bleeding control in different situations has always been an important topic of multidisciplinary research.

Hemostatic techniques and materials with excellent properties are essential for saving lives and reducing adverse side effects. Traditional hemostatic materials (e. g. tourniquets, hemostatic gauze, bandages, and etc.) have been widely used for rapid hemostasis of superficial wounds (Leonard et al., 2016; Montaser et al., 2020; Farahani and Shafiee, 2021). However, as to injuries with intracavitary hemorrhage or involving non-compressive vital tissues/organs (e. g. brain tissue, spinal cord, fragile organs, and etc.), traditional techniques are difficult to meet the demand for rapid and effective hemostasis (Jones et al., 2018; Montaser et al., 2020). Moreover, gauze or bandages need to be completely removed after hemostasis because of their non-degradability, which may cause secondary injury, delayed healing, and additional pain (Hoekstra et al., 2002). The exploration of advanced hemostatic materials including biodegradable gauze, sponges, powders, sprays, and hydrogels have been ongoing. In recent years, the rapid development of hydrogel-based biomaterials for hemostatic and wound healing have led to the emergence of solutions to clinical hemostatic challenges.

Hydrogel-based biomaterials have shown many advantages compared with traditional hemostatic methods. Based on their injectability and flowability, hydrogels can be applied to a variety of irregular wounds and intracavity injuries, which is meaningful for rapid and effective hemostasis. Furthermore, the excellent biocompatibility and biodegradability ensure the safety of hydrogel-based biomaterials for *in vivo* applications and enhance their ability to promote wound healing. Other properties such as drug delivery, self-healing, self-growth, and stimulation response can be tailored as needed, thus endowing the materials with additional therapeutic functions (Khunmanee et al., 2017; Vakalopoulos et al., 2017; Wang et al., 2019b; Hashemnejad and Kundu, 2019; Matsuda et al., 2019; Xue et al., 2019). Nevertheless, further clinical application of hydrogel-based biomaterials is constrained by certain shortcomings, including weak wet surface adhesion, poor mechanical properties, delayed *in situ* gel formation, and uncontrollable degradation (Bu et al., 2017; Li et al., 2017; Zhu et al., 2018a; Rao et al., 2018).

This review demonstrates a brief introduction of the physiological mechanisms of hemostasis, and focuses on the natural-derived polysaccharide- and protein-based hemostatic hydrogels in terms of preparation strategies, physicochemical properties, hemostatic and wound-healing abilities, as well as recent advances for clinical applications.

## Biological Mechanisms of Hemostasis

The physiological mechanism of hemostasis in the human body is a complex and dynamic process consisting of a series of spatiotemporal reactions. In healthy conditions, vascular endothelial cells secrete a series of anticoagulant factors (heparin-like molecules, thrombomodulators, nitric oxide and prostacyclin) to avoid blood clotting (Hickman et al., 2018). When tissue injury leads to vascular rupture and bleeding, the body will respond rapidly by promoting reactive local vasoconstriction to reduce blood loss, while at the same time promoting the secretion of clotting factors and related proteins and mobilizing the action of platelets (Ruggeri and Mendolicchio, 2007). The damage to vascular endothelium exposes the subendothelial collagen, which attracts platelets for adhesion, and the formation of platelet thrombi is initiated (Versteeg et al., 2013). Normally, platelets adhere to collagen with integrin α2β1 and glycoprotein (GP) VI as the two main receptors, while adhesion under high-shear conditions is enhanced with the help of GPIb-V-IX receptor complexes and von Willebrand factor (vWF) (Wang et al., 2018). The adhesion process promotes platelet activation and aggregation at the bleeding site, enabling rapid platelet thrombus formation, which is known as “primary hemostasis” (Boulton, 2006; Varga-Szabo et al., 2008; Makris et al., 2011). In tandem, the coagulation cascade, known as “secondary hemostasis”, is initiated by both endogenous and exogenous pathways (Roberts et al., 2006). The exogenous pathway is activated by the exposure of tissue factor in the blood, while the endogenous pathway is triggered by the exposure of factor XII to foreign substances with negative charges on the surface, both of which ultimately lead to the activation of coagulation factor X (Hoffman, 2003). Activated factor X converts prothrombinogen into thrombin, which in turn rapidly converts fibrinogen into fibrin monomers, and the monomers could aggregate to form a fibrin network structure (Gale, 2011). Finally, activated factor XIII immobilizes platelet thrombi and other blood components at the bleeding site and forms the final clot by inducing intrafibrillar cross-linking (Morrissey, 2012; Chan et al., 2015). Therefore, hemostatic biomaterials should focus on mimicking and exploiting the complex coagulation mechanisms described above in order to achieve more rapid and effective hemostatic efficacy.

## Polysaccharide-based Hemostatic Hydrogels

Polysaccharides are widely present in the natural world as reproducible substances. They have excellent biocompatibility and biodegradability, and in some cases exhibit the ability to promote hemostasis, accelerate wound healing, and antibacterial properties (Yang et al., 2017; Khan and Mujahid, 2019; Chen et al., 2020). However, the deficiency of adhesion strength and mechanical properties constrains the application of polysaccharides being used for the preparation of hydrogel adhesives (Zhong et al., 2021). Therefore, chemical modification and cross-linking with various of performance-enhanced materials has become widely accepted programs (Zhang et al., 2021).

### Chitosan

**FIGURE 1:**
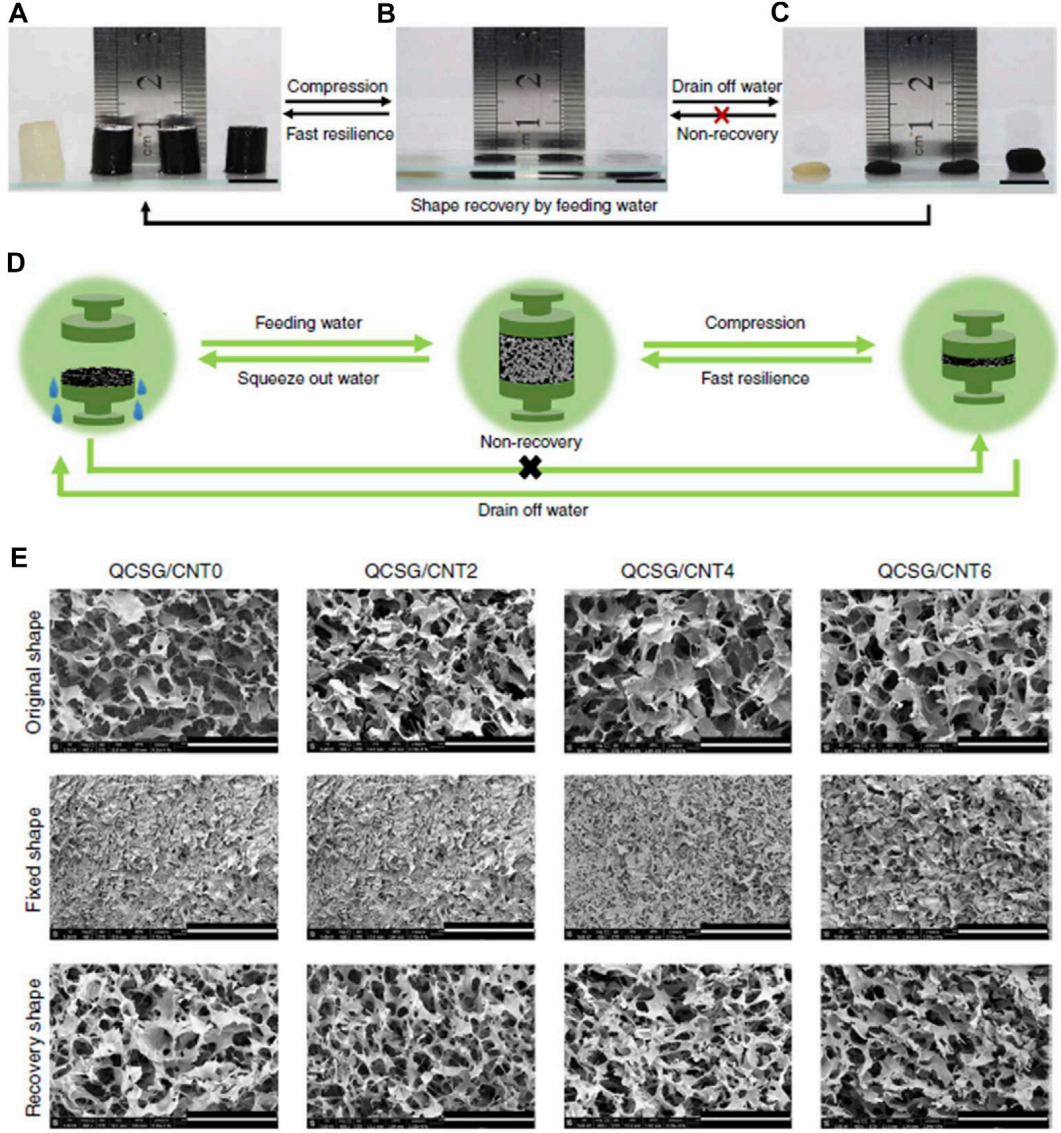
Shape memory properties of the cryogels. **(A–C)** Fast resilience and macroscopical shape memory property of the cryogels. Scale bar: 1 cm. **(D)** Schematic representation of the shape memory mechanism of the cryogel. **(E)** Microtopography of the cryogels in original state, shape-fixed state and shape recovery state after fixing. Scale bar: 400 μm. Reproduced from (Zhao et al., 2018) with permission from Copyright 2018 Springer.

The excellent biocompatibility and degradability, as well as the hemostatic and antibacterial properties reported in previous studies, make chitosan an ideal candidate for hemostatic biomaterials (Jayakumar et al., 2011; Malette et al., 1983; Chou et al., 2003; Cheung et al., 2015). Wang et al. prepared a novel photo-crosslinked chitosan hemostatic hydrogel by introducing two crosslinking mechanisms (photo-induced carbon-carbon double bond crosslinking; catechol-Fe^3+^ chelation), and two chitosan polymer networks (catechol-modified methacryloyl chitosan; methacryloyl chitosan) (Wang et al., 2020a). The hydrogel not only showed hemostatic properties in a mice liver hemorrhage model, but also exhibited excellent antimicrobial and healing-promoting abilities through Staphylococcus aureus-infected full-thickness skin wound model. Zhao et al. prepared an injectable multifunctional nanocomposite cryogel for hemostasis and healing of incompressible wounds. The cryogel based on carbon nanotubes (CNT) and glycidyl methacrylate functionalized quaternized chitosan (QCSG) showed better coagulation ability, higher blood cell and platelet adhesion and activation than gelatin sponge and gauze, and excellent hemostatic performance in a rabbit liver defect lethal incompressible hemorrhage model. It even showed better hemostasis than Combat Gauze in a standardized round liver hemorrhage model (Zhao et al., 2018) (Figure 1). In another study by Zhao et al., an antimicrobial electroactive injectable hydrogel dressing was discussed. Hydrogels with the main components of quaternized chitosan-g-polyaniline and benzaldehyde group functionalized polyethylene glycol (PEG)-co-poly (glycerol sebacate) was able to promote wound healing, increase granulation tissue thickness and collagen deposition in a full-thickness skin defect model. In addition, the hydrogel dressing could rely on its self-healing ability to extend its service life, which may provide significant convenience for potential clinical applications (Zhao et al., 2017).

**FIGURE 2:**
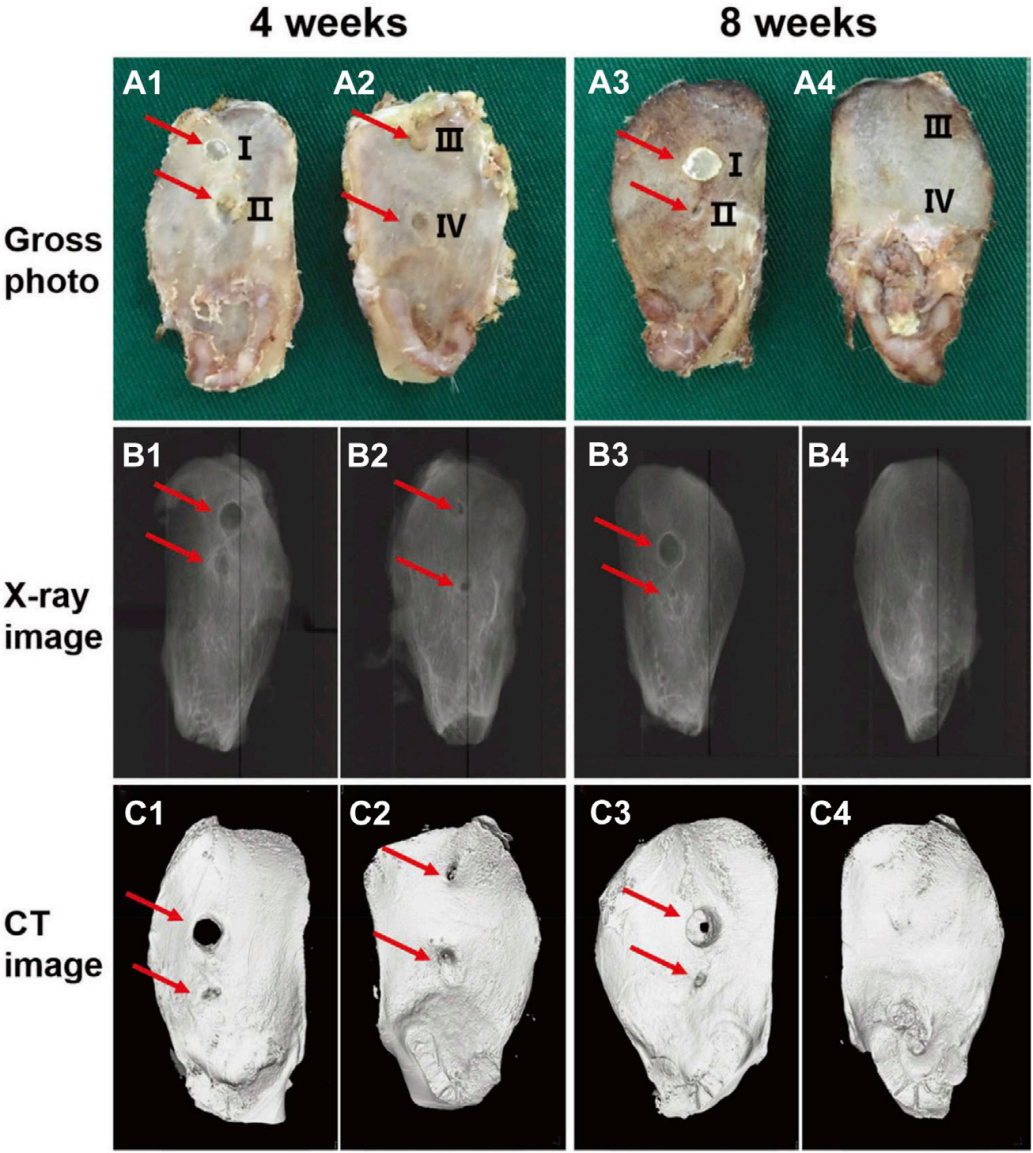
Representative gross photograph **(A1–A4)**, radiophograph **(B1–B4)**, and micro-CT scan images **(C1–C4)** of the ilium bone defects of New Zealand rabbits receiving no treatment (III) or treated with bone wax (I), CHI-C solution (II), or CHI-C/DACNC hydrogel (IV) after 4 and 8 weeks of surgery. The CHI-C/DACNC group shows more abundant bone formation than other groups. Reproduced from (Huang et al., 2021a) with permission from Copyright 2021 Wiley.

Qu et al. prepared a self-healing injectable wound dressing with quaternized chitosan (QCS)-based hydrogel (Qu et al., 2018). The dressing showed tensile and compressive properties comparable to those of human skin and could be used for joint skin injuries. Curcumin loading enabled the dressing to demonstrate favorable antioxidant capacity and pH-responsive release profile, which significantly accelerated wound healing and upregulated vascular endothelial growth factor (VEGF) in a full-thickness skin wound model. To manage bone bleeding and bone defects derived from trauma or bone tumor resection, Huang et al. introduced a multifunctional hydrogel fabricated with catechol-conjugated chitosan (CHI-C) and dialdehyde cellulose nanocrystal (DACNC) (Huang et al., 2021a). After injection into the bone defect area, the hydrogel could be coagulated *in situ* within 2 min. The ability of this hydrogel to stop bleeding and promote bone regeneration was demonstrated in a rabbit iliac bone defect model (Figure 2). In addition, Eugene et al. prepared a kind of chitosan-PEG-tyramine hydrogel and explored its performance as effective tissue adhesive. Using sutures, fibrin glue and cyanoacrylate as controls, the hydrogels showed rapid glue-forming ability within 5 s and better wound healing in a rat skin incision model (Lih et al., 2012).

### Hyaluronic Acid

**FIGURE 3:**
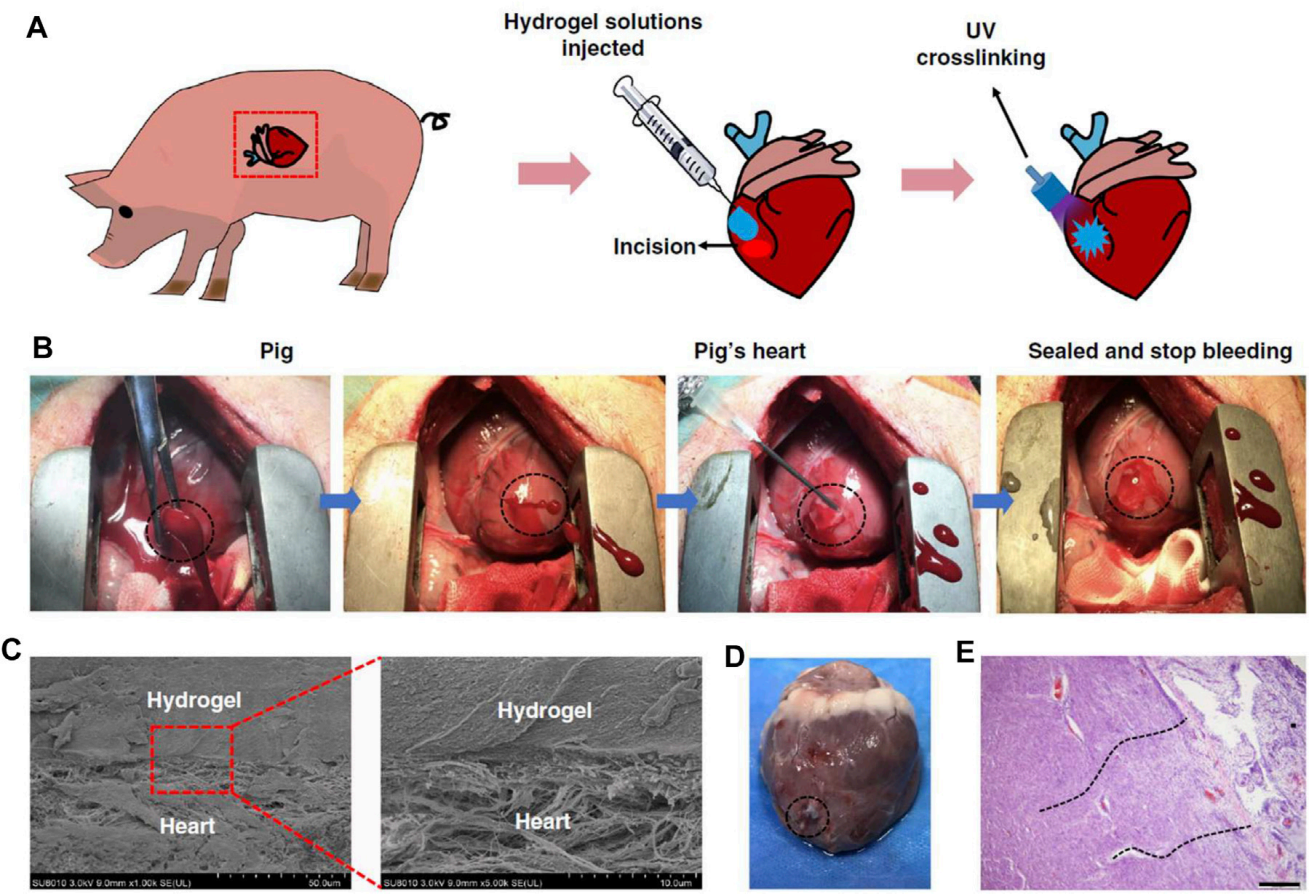
Hemostatic properties of the matrix gel in a pig cardiac puncture injury model. **(A)** Schematic diagram of the surgical procedure. **(B)** Gross view of the rapid hemostasis and sealing following cardiac puncture injury. **(C)** Scanning electron micrographs of the interface between the pig heart puncture wound and the hydrogel. Scale bar: 50 μm (left plates); 10 μm (right plates, enlarged). **(D)** Images of a heart autopsy following killing after two 2 weeks of postoperative recovery, the hydrogel still adhering to the wound. **(E)** Tissue staining images of the interface between pig heart cardiac tissue and the matrix gel, after 2 weeks of postoperative recovery. Scale bar: 200 μm (*n* = 4). Reproduced from (Hong et al., 2019) with permission from Copyright 2019 Springer.

In response to the weak adhesion of existing hemostatic agents to moist and mobile tissues, an impressive study on hydrogel adhesives was conducted by Hong et al. (2019) The design of this hydrogel system with gelatin and hyaluronic acid (HA) as the main components was inspired by the extracellular matrix. To verify the powerful hemostatic properties of the hydrogel, the authors selected porcine carotid artery and heart hemorrhage models, which placed high demands on the wet surface adhesive ability, mechanical properties, and rapid gelling and fixation ability of the hemostatic material. Encouragingly, the hydrogel successfully sealed the high-pressure hemorrhage from a 4- to 5-mm carotid artery incision and a 6-mm diameter cardiac perforation (Figure 3). Zhu et al. designed a hemostatic and anti-infective hydrogel with sustained drug release capability (Zhu et al., 2018b). The composite material was consisted of aminoethyl methacrylate HA (HA-AEMA), methacrylated methoxy PEG (mPEG-MA) hybrid hydrogels and chlorhexidine diacetate (CHX)-loaded nanogels. *In vivo* hemostasis and wound healing were evaluated using mice liver hemorrhage and full-thickness skin wound models. The results confirmed the ability of the composite hydrogel to rapidly stop bleeding, accelerate wound healing, and prevent infection.

Luo et al. introduced a HA/collagen hemostatic hydrogel that was prepared *in situ* on the tissue surface. *In vitro* experiments revealed that the hydrogel was stronger than fibrin glue in terms of rupture strength, and a rat liver hemorrhage model showed its hemostatic ability comparable to that of fibrin glue (Luo et al., 2019). As a novel procoagulant, inorganic polyphosphate (PolyP) stored in platelet-dense granules is receiving increasing attention (Ruiz et al., 2004). PolyP has been reported to promote coagulation through four pathways: 1) initiation of the coagulation cascade reaction; 2) activation of coagulation factor V; 3) stabilization of fibrin clots; and 4) facilitation of factor XI feedback activation by thrombin (Müller et al., 2009; Mutch et al., 2010; Choi et al., 2011). Sacoda et al. prepared a hemostatic hydrogel with HA and PolyP. Biocompatibility of the composite hydrogel *in vitro* and *in vivo* was demonstrated by viability assays of three cell lines (macrophages, fibroblasts and mesothelial cells) and by intraperitoneal and subcutaneous injections. In addition, the hydrogels showed similar hemostatic effects compared to fibrin glue in a mise liver hemorrhage model (Sakoda et al., 2018).

### Alginate

**FIGURE 4:**
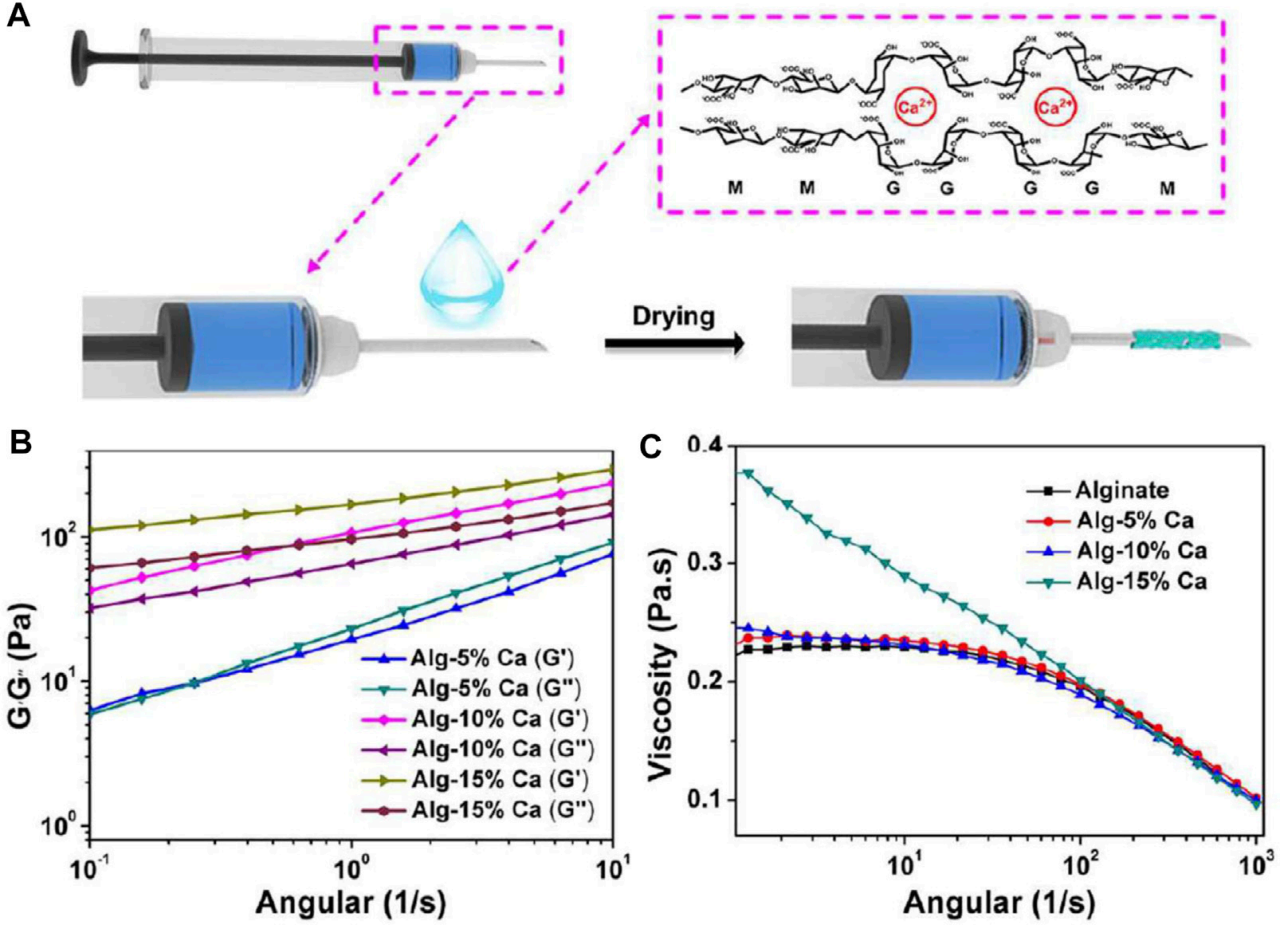
Preparation of the hemostatic needles with the Alg-Ca coatings. **(A)** Schematic illustration of the preparation of hemostatic hydrogel coatings on syringe needles. **(B)** Rheological analysis of Alg-5% Ca, Alg-10% Ca, and Alg-15% Ca hydrogel films after exposure to serum. **(C)** The viscosity of alginate, Alg-5% Ca, Alg-10% Ca, and Alg-15% Ca precursor solutions. Reproduced from (Ren et al., 2020) with permission from Copyright 2020 Elsevier.

Alginate is an anionic polysaccharide with a strong water absorption capacity (Aderibigbe and Buyana, 2018). It also can activate the coagulation cascade reaction and accelerate platelet aggregation after cross-linking with Ca^2+^, thus accelerating hemostasis (Lee and Mooney, 2012). Namitha et al. constructed a supramolecular hydrogel scaffold with alginate/poly (N-vinyl caprolactam), followed by ionic cross-linking with Ca^2+^ and tannic acid (TA) to prepare a pH and temperature dual responsive supramolecular hydrogel (Preman et al., 2020). Hydrogels showed good mechanical properties, and their pore size could be regulated by changing the ratio of polymers. The proper porosity promoted the migration of fibroblasts, the exchange of nutrients and the absorption of exudate. By coating syringe needles with hemostatic hydrogels, Ren et al. prepared a kind of hemostatic needles. The Alginate-based hydrogel (Alg-Ca) would detach from the surface of the needle and stay at the puncture site, preventing further bleeding (Ren et al., 2020). The hemostatic capability of the hydrogel-coated needles in the blood vessel puncture was tested by models of rat external jugular vein and rabbit ear vein, and in viscera puncture was tested by rat kidney and liver. In addition, the hemostatic effect was also investigated in the external jugular vein of hemophilic mice (Figure 4).

Oxidized alginate (OA), a dialdehyde derivative of alginate, with a large number of aldehyde groups on its surface which can form Schiff base bonds with the amine groups of histones, thus greatly enhancing the adhesion properties(Reakasame and Boccaccini, 2018). Song et al. prepared dopamine-alginate oxide hydrogels (Dopa-OA) by introducing dopamine into the OA molecular chain in order to further enhance the adhesion properties of OA hydrogels. Meanwhile, polyallylamine (PAA) was selected as the internal structural polymer to improve the mechanical properties (Song et al., 2019). The composite hydrogel could be rapidly gelled in 5–10 s and showed good hemostatic properties in a mice liver hemorrhage model. In the study by Kong et al., a series of hydrogel dressings based on N-carboxyethyl chitosan (CEC) and OA were developed for wound healing (Kong et al., 2021). In a mice full-thickness skin wound model, these hydrogels could significantly promote the healing of infected wounds. Liu et al. developed composite hydrogels with tissue factor-integrated liposomes combined with alginate. Fluorescence measurements showed that the proteoliposomes were uniformly distributed in the alginate matrix and remained intact even after release into simulated body fluids, which exhibited excellent hemostatic properties (Liu et al., 2020).

### Cellulose

Cellulose is a major component of plant cell walls and can also be produced by certain microorganisms (Mano et al., 2007). Due to the cost-effective production and the excellent mechanical properties, cellulose has been widely researched in biomedical area (Xiao et al., 2019; Alven and Aderibigbe, 2020). In the study by Huang et al., a hydrogel (CMC/PEG-BA) based on carboxymethyl cellulose (CMC) and a four-armed PEG capped with benzaldehyde (PEG-BA) was developed (Huang et al., 2016). At the same crosslink density, the four-armed PEG network of CMC/PEG-BA was more resistant to fracture than the normal two-armed PEG. After application to rabbit liver incisions, CMC/PEG-BA hydrogel showed good hemostatic ability compared to sterile gauze. Histological evaluation revealed that sterile gauze resulted in a large gap between the wound interfaces, while CMC/PEG-BA was able to trap red blood cells and fill the injury space. Deng et al. made a composite hydrogel by combining fenugreek gum with cellulose through hydrogen bonding in order to impart better mechanical properties to the hemostatic hydrogel (Deng et al., 2020). Based on a porous fiber network structure, the hydrogel can rapidly absorb wound exudate and demonstrated the ability to stop bleeding and promote wound healing through mice liver hemorrhage and skin defect models.

**FIGURE 5:**
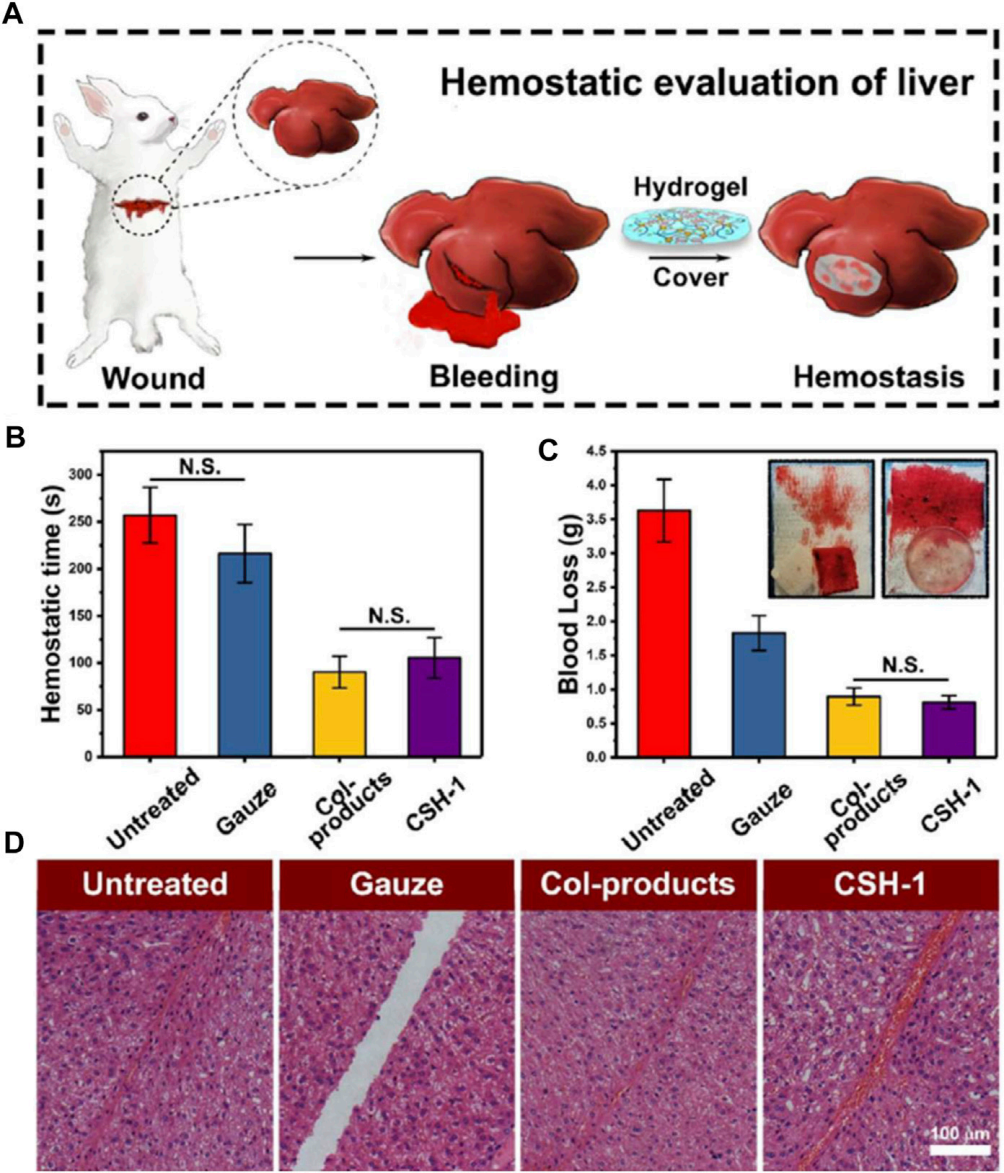
Hemostatic evaluation of cellulose/SF hydrogels (CSHs). **(A)** Surgery scheme, showing that the hemostatic assay was performed with rabbit liver (rabbit experimental number = 4). **(B)** Hemostatic time from the assay. **(C)** Blood loss assay. **(D)** HE staining of the incisions (the scale bar was 100 μm), showing the CSH-1 activated hemocytes’ aggregation in the wounded sites in comparison with the untreated, gauze samples, and Col-products. For hemostatic time and blood loss test, the results were calculated as mean ± SD, *n* = 4. Reproduced from (Wang et al., 2020b) with permission from Copyright 2020 American Chemical Society.

The study by Wang et al. demonstrated a CO_2_-mediated chemical cross-linking strategy that avoids the use of toxic cross-linking agents to make biocompatible, mechanically strong double-network cellulose/silk fibroin hydrogels (CSH). Through a rat full-thickness skin injury model and a rabbit liver hemorrhage model, the authors verified the hemostatic and wound healing potential of this dual-network hydrogel (Figure 5) (Wang et al., 2020b). Tavakoli et al. developed a novel Kappa carrageenan (κCA)-coated cellulose nanofiber (CNF)/starch nanocomposite hydrogel for hemostasis. The κCA coating imparted higher mechanical strength and lower swelling and degradation rates to the hydrogel, while maintaining the good biocompatibility and hemostatic ability of the starch and cellulose matrices, making κCA-coated starch/CNF hydrogel an ideal candidate for hemostatic applications (Tavakoli et al., 2021). Mendes et al. proposed a porous network hemostatic cryogel based on platelet lysate (PL) and aldehyde-functionalized cellulose nanocrystals (a-CNC) covalently cross-linked. Upon immersion into blood, PL-CNC cryogels showed more powerful absorption capacity compared to commercial hemostatic gelatin sponges. Impressively, the cryogel can release biomolecules to increase stem cell proliferation and migration and down-regulate the expression of fibrinolytic process markers (Mendes et al., 2020).

## Protein-Based Hemostatic Hydrogels

Protein-based biomaterials are of wide interest in the biomedical field due to their high mechanical strength, biocompatibility, biodegradability, and flexibility in structure-directed mechanics (Löwik et al., 2010; Woolfson and Mahmoud, 2010; Silva et al., 2014; Nguyen et al., 2018; Zhou et al., 2018). To date, protein-based hydrogels have been extensively developed and are considered as one of the ideal candidates for hemostasis and promotion of tissue healing therapy (Maham et al., 2009; Huang et al., 2018). Collagen, silk, and elastin are common structural proteins for the preparation of protein-based hemostatic biomaterials (Altman et al., 2003; Almine et al., 2010; Chattopadhyay and Raines, 2014).

### Gelatin

**FIGURE 6:**
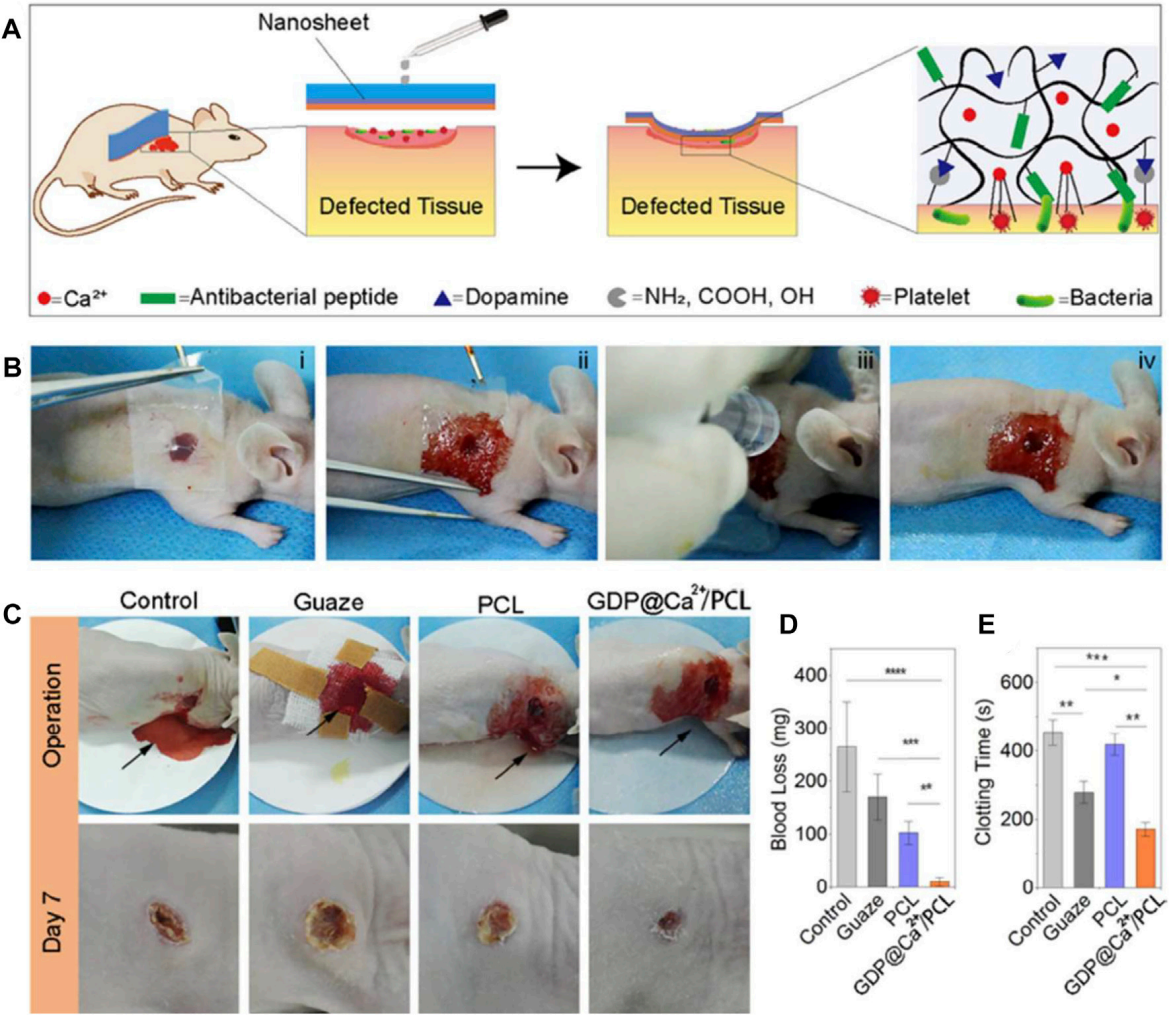
The hemostatic and sealing performances of nanosheets on the dorsal skin of a nude mice model. **(A)** Schematic of using a nanosheet to heal skin wounds with bleeding and infection. **(B)** The operation process of a GDP@Ca^2+^/PCL nanosheet on a wound. **(C)** Images of wound healing after treatment with gauze, PCL and the GDP@Ca^2+^/PCL nanosheets, with an untreated wound serving as the control group. **(D,E)** The blood loss **(D)** and clotting time **(E)** evaluations of the samples. Reproduced from (Xuan et al., 2020) with permission from Copyright 2020 Elsevier.

Gelatin, derived from partial hydrolysis of collagen, has been well reported as a highly promising candidate for biomaterials. However, its further applications are facing some challenges, including low shape stability, rapid degradation profile, and poor mechanical properties (Wang et al., 2014; Sun et al., 2020). Xuan et al. designed a flexible antimicrobial hemostatic dressing with two layers of dopamine/antimicrobial peptide modified gelatin (GDP) and Ca^2+^ ions (GDP@Ca^2+^) to provide antimicrobial and hemostatic properties, and another was composed of polycaprolactone (PCL) to provide mechanical strength (Xuan et al., 2020). *In vivo* evaluation of the bilayer dressing with mice dorsal skin and liver models further demonstrated that the dressing successfully adhered to the tissue surface in a wet bleeding environment, promoting healing while maintaining antimicrobial action for up to 2 weeks (Figure 6).

**FIGURE 7:**
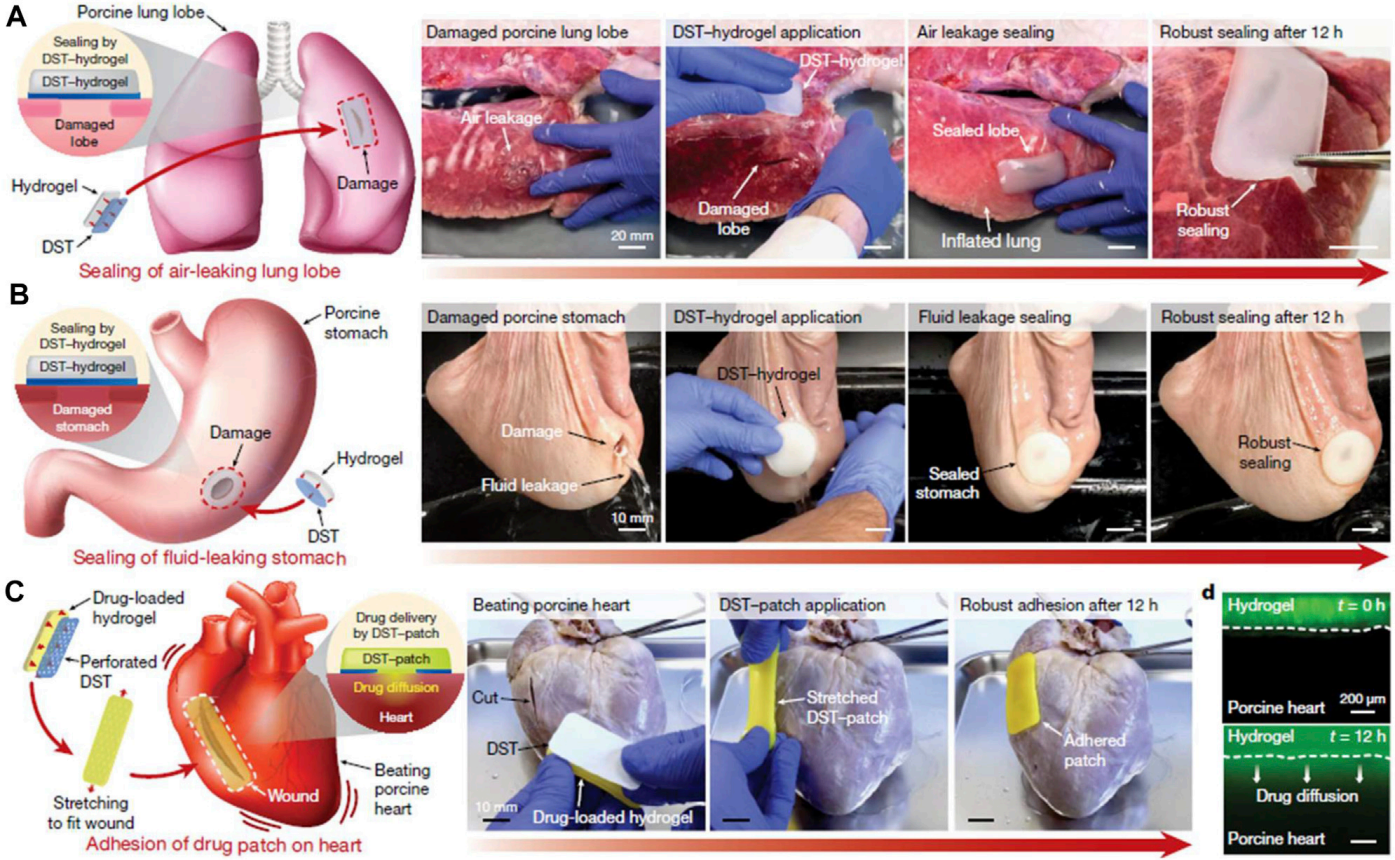
Potential applications of the DST. **(A)** Sealing of an air-leaking lacerated *ex vivo* porcine lung lobe by a hydrogel patch adhered with the DST. **(B)** Sealing of a fluid-leaking *ex vivo* porcine stomach by a hydrogel patch adhered with the DST. **(C)** DST-mediated adhesion of a drug-loaded patch on a beating *ex vivo* porcine heart with a cut. **(D)** Diffusion of a mock drug (fluorescein) from a DST-adhered drug patch into the *ex vivo* porcine heart tissue over time. Reproduced from (Yuk et al., 2019) with permission from Copyright 2019 Springer.

Tang et al. constructed a three-dimensional network hydrogel, consisting of methacrylated hyaluronan-polyacrylamide, silver nanoparticles and gelatin (Tang et al., 2020). The adhesive was capable of sustained release of silver ions and thus possessed broad-spectrum antimicrobial activity. In a rat wound infection model, the adhesive showed superior ability to promote wound healing. Yuk et al. reported a dry double-sided tape (DST) for tissue adhesion in wet environment. The tape had a thin hydrogel surface made from a combination of gelatin/chitosan and crosslinked poly (acrylic acid) grafted with N-hydrosuccinimide ester (Yuk et al., 2019). The adhesive mechanism relied on the ability to remove interfacial fluids from tissue surface, thus allowing rapid temporary cross-linking of the material to the tissue surface. The subsequent interfacial covalent cross-linking further improved the adhesion strength and stability. The authors showed through a series of *ex vivo* organ rupture models (lung, stomach, heart and intestine) that DST could achieve strong adhesion to a variety of wet dynamic tissue surfaces within seconds (Figure 7).

Gaharwar et al. reported a nanocomposite hemostatic hydrogel consisting of gelatin and synthetic silicate nanoplates (Ak et al., 2014). The nanocomposite reduced blood clotting time by 77% and formed stable clot-gel systems *in vitro*, and *in vivo* testing demonstrated its ability to promote hemostasis in fatal liver lacerations. In a study by Liu et al., double network hydrogels were prepared using TA and gelatin methacrylate (GelMA), and the changes in morphology and mechanical properties of hydrogels were explored at different TA concentrations and treatment times (Liu et al., 2018). The results showed that the mechanical properties and adhesion capacity of GelMA-TA hydrogels were significantly improved.

### Silk

Silk is a common natural fibrous protein, usually produced by arthropods (e.g., silkworms, bees, spiders, etc.), with unique physicochemical properties (Ma et al., 2018; Tomeh et al., 2019). Low molecular weight Silk fibroin (SF) has been shown to have a definite hemostatic ability (Lei et al., 2016). Huang et al. prepared hemostatic microspheres with different degrees of surface roughness by cross-linking sodium alginate and SF in order to accelerate the blood coagulation. The results showed that when the volume ratio of SA to SF was adjusted to 2:1 (SF/SA2), the surface of the microspheres was the roughest, and thus more red blood cells could be aggregated and the coagulation speed of the hemostatic agent was apparently accelerated (Huang et al., 2021b). Shefa et al. constructed a hydrogel scaffold using oxidized cellulose nanofibers and SF, and explored the effect of thrombin loading on the hemostatic properties of this scaffold. Ultimately, the thrombin-loaded hydrogel scaffold was shown to have good hemostatic ability through the rabbit ear artery as well as the rat severed tail and liver hemorrhage models (Shefa et al., 2019). In the study by Serban et al. SF and PEG were used in the research of hemostatic adhesives. The composite hydrogel can be rapidly formed within seconds by chemical cross-linking and exhibits lower swelling rates and longer degradation times (Serban et al., 2011).

Referring to the chemical composition and hierarchical nanostructure of mussel foot proteins, Bai et al. introduced TA into SF and developed a hemostatic hydrogel sealant with excellent wet adhesion properties (Bai et al., 2019). When being used to seal ruptured bleeding tissue *in vivo*, the composite hydrogel demonstrated rapid and effective hemostasis, while maintaining good biocompatibility and biodegradability, as well as outstanding antimicrobial activity. The hydrogel was able to adhere firmly to the tissue surface even in a wet dynamic environment, which undoubtedly increased the reliability of its hemostatic effect. Likewise inspired by mussel adhesion proteins, Burke et al. designed a mechanically enhanced catechol-functionalized filamentous protein hydrogel (Burke et al., 2016). The potential advantage of catechol-SF over the available water-soluble catechol adducts based primarily on the PEG was that the catechol-SF were water processable and its hydrophobicity resulted in lower swelling *in vivo* than that of catechol-PEG. Sen et al. explored the role of hydrogel scaffolds synthesized from SF and polyurethane in diabetic wound healing (Sen et al., 2020). As a wound dressing, the composite material with excellent exudate absorption and broad-spectrum antibacterial ability could significantly promote the healing of chronic hyperglycemic wounds.

### Elastin

Elastin is an important protein component of the extracellular matrix (ECM) in tissues such as the vascular system, skin, and lung (Vindin et al., 2019). It is once regarded as an expected source of biomaterials. However, the insolubility and structural stability of elastin hamper its mass production and further research (Kielty et al., 2002). Accordingly, soluble tropoelastin (precise replicas of the natural elastin precursor) as well as elastin-like polypeptides (ELP) have emerged as candidates for elastin (Wise and Weiss, 2009; Kozel and Mecham, 2019; Reichheld et al., 2019). Annabi et al. designed a highly elastic hydrogel sealant with tunable adhesion properties for surgical applications. The methacrylate-substituted tropoelastin (MeTro) hydrogel was obtained by photo-crosslinking the recombinant human protein tropoelastin (Annabi et al., 2017a). After MeTro hydrogel was used to seal a series of *in vivo* incision models (rat arteries, lungs, and porcine lungs), all animals were able to survive without functional abnormalities during the observation period. Based on the above work, Annabi et al. reported a sprayable, elastic composite hydrogel composed of two ECM-derived biopolymers (GelMA and MeTro) for the treatment of chronic wounds (Annabi et al., 2017b). Interestingly, the physical properties of hydrogel can be fine-tuned by varying the MeTro/GelMA ratio and the final polymer concentration. Moreover, loading of antimicrobial peptides conferred broad-spectrum antimicrobial ability to the composite hydrogel.

Brennan et al. prepared an adhesive with underwater adhesion and “smart” environmental response behavior. The adhesive is constructed with an ELP from *Escherichia coli* and modified with 3,4-dihydroxyphenylalanine (DOPA), which can coacervate in response to environmental factors such as temperature, pH and salinity (Brennan et al., 2017). Compared to commercially available fibrin glues, this adhesive exhibits significantly higher adhesion strength in dry, wet and even submerged environments. In the study by Desai et al. flexible hydrogel adhesive was prepared, similarly based on the chemical modification of ELP by dopamine (Desai et al., 2020). The hydrogel exhibited a stable swelling rate at 37°C under aqueous conditions. In addition, the adhesive strength of the flexible adhesive was revealed through tensile pull-off and lap-shear testing on porcine skin. In another attempt to overcome adhesion challenges in the underwater environment, Narayanan prepared a kind of tropoelastin-like self-coacervating polyesters that could mimic the self-coacervation and environmental stability of a mussel adhesive protein (Narayanan et al., 2020). This smart material demonstrates the potential for rapid underwater adhesion applications.

## Conclusion and Outlook

When facing a hemorrhagic injury, the body’s inherent hemostatic mechanisms become insufficient to rapidly stop blood loss, and certain materials and techniques are often needed to achieve rapid hemostasis (Neubauer and Zieger, 2021). Traditional hemostatic methods (tourniquets, gauze, bandages, etc.) are mainly relied on the physical blockage and limited activation of the coagulation system, which can hardly achieve effective hemostasis and may instead face additional problems such as secondary injury, immune rejection and infectious risk (Hagemann et al., 2013; Hickman et al., 2018). Commercially available hemostatic materials such as porcine-derived fibrin sealant and gelatin matrix have been approved for clinical application, but adhesion and mechanical deficiencies combined with high cost and risk of disease transmission greatly limit their clinical efficacy, especially in acute or severe bleeding situations. Therefore, modern medicine needs a revolution in hemostatic techniques urgently to address the numerous medical risks associated with excessive blood loss. Hydrogels derived from polysaccharides and proteins are biocompatible, biodegradable, non-immunogenic, and can provide powerful hemostatic ability by promoting, enhancing, compensating, or mimicking the natural mechanisms of hemostasis (Ryu et al., 2015; Zhang et al., 2015; Hu et al., 2019; Rizzo and Kehr, 2021). Although research on polysaccharide- and protein-based hydrogels for hemostasis continues to gain momentum, there are still critical issues to overcome before they can be translated for clinical applications. The challenge lies mainly in the difficulty of achieving the harmonization between biosafety, hemostatic effectiveness, and practical feasibility.

It is of great significance to define the key properties that an ideal hemostatic hydrogel-based biomaterial should have and thus guide the future research. When being applied to the human body, hydrogel-based materials must primarily ensure biosafety. In this regard, biocompatibility, biodegradability, non-toxicity, and non-immunogenicity should be the prerequisites for the application of ideal hemostatic hydrogels. On the other hand, the effectiveness of hemostasis needs to be considered, which requires biomaterials to possess not only superior hemostatic ability, but also outstanding physical and chemical properties to meet the needs of specific conditions, for example, a wet, dynamic, irregular wound. Therefore, reliable biocompatibility, biodegradability, mechanical strength, wet surface adhesion, viscoelasticity, and fatigue resistance are necessary for becoming ideal hemostatic hydrogel-based biomaterials. In addition to biosafety and hemostatic effectiveness, practical feasibility is equally significant and easily ignored. Reduced production costs, simplified preparation processes, ease of handling, and convenient preservation are important prerequisites for clinical applications. In addition, hemostatic hydrogels need to be individually designed to meet the requirement of complicated clinical situations. For example, stronger wet surface adhesion is required in the face of abnormal coagulation due to chronic disease. Hemostasis around nerve tissue imposes stringent requirements on the control of swelling rates. hemostasis of internal organs requires hydrogels that can resist specific enzyme without enzymatic breakdown before complete hemostasis is achieved. Other properties such as wound healing promotion, anti-inflammatory and antimicrobial capabilities are also part of the hemostasis requirements in certain circumstances and need to be addressed on a situation-specific basis. There are no uniform standards for the adhesion and mechanical strength of ideal hemostatic materials, which may be due to differences in application requirements (skin, organ, arterial or cardiac surface hemostasis) and hemostatic mechanisms (physical sealing, coagulation mechanisms). In general, the ideal hemostatic hydrogel should have an adhesion strength of at least tens to hundreds KPa.

Natural-derived polysaccharide- and protein-based hydrogels have the advantages of excellent biosafety, however, their hemostatic effectiveness are often compromised by insufficient mechanical and adhesion strength (Zia et al., 2015; Alven and Aderibigbe, 2020; Graça et al., 2020; Zhang et al., 2020). Further research is expected to improve wet surface adhesion, pressure-resistant intensity and rapid gelation ability through multiple methods, including chemical modifications, cross-linking with functionalized components, advanced preparation techniques, and thus facilitating the transformation of hemostatic biomaterials from laboratory research to clinical applications. Further research should be devoted to enhancing hemostatic efficacy through a variety of methods. However, in order to enhance the mechanical properties, many of the current hemostatic hydrogels are prepared by complex synthetic routes and often with the help of non-biocompatible synthetic polymers, a process that is highly susceptible to reduced biosafety. Therefore, to ensure biosafety, the reaction steps should be simplified as much as possible, while avoiding the addition of components with poor biocompatibility. The prepared materials should undergo comprehensive safety verification, including the metabolic pathways of degradation products as well as a toxicological testing. Simplified chemistry reactions also facilitate control production costs, and materials consisting of simple ingredients approved by regulatory agencies are more likely to enter clinical trials. Future research on the enhancement of hemostatic ability can be achieved either by increasing the adhesion and mechanical properties of hydrogels or by enhancing the physiological coagulation process. If the rapid formation of blood clots can be promoted to reinforce the hemorrhage seal, the requirements for the adhesive and mechanical properties of the hydrogels can be relatively reduced. This process can be achieved with the loading of key coagulation factors or proteins.

In conclusion, by summarizing previous preclinical studies, we discussed the natural-derived polysaccharide-based (chitosan, HA, alginate, cellulose) and protein-based (gelatin, silk, elastin) hemostatic hydrogels in terms of preparation strategies, physicochemical properties, hemostatic and wound-healing abilities. These life-saving materials are expected to revolutionize civilian or military bleeding control options, improve the quality of treatment, and ultimately lead to a radical change in hemostasis technology.
